# Obesity Trends among Asthma Patients in the United States: A Population-based Study

**DOI:** 10.5334/aogh.2420

**Published:** 2019-01-22

**Authors:** Maria Fernanda Lurbet, Belen Rojano, Stacey-Ann Whittaker Brown, Paula Busse, Fernando Holguin, Alex D. Federman, Juan P. Wisnivesky

**Affiliations:** 1 Division of General Internal Medicine, Department of Medicine, Instituto Universitario CEMIC, Ciudad Autónoma de Buenos Aires, AR; 2 Division of General Internal Medicine, Icahn School of Medicine at Mount Sinai, NY, US; 3 Division of Pulmonary, Critical Care, and Sleep Medicine, Icahn School of Medicine at Mount Sinai, NY, US; 4 Division of Allergy and Immunology, Icahn School of Medicine at Mount Sinai, NY, US; 5 Division of Pulmonary Medicine, University of Colorado Denver, Denver, CO, US

## Abstract

**Background:**

Obesity is strongly associated with worse asthma control and poorer quality of life. The current obesity epidemic has reached historically high levels, with an estimated prevalence rate of 37% in the general United States (US) population. However, less is known about trends in the prevalence of obesity among individuals with asthma or which sociodemographic groups are at higher risk for increased weight.

**Methods:**

The study was conducted with data from the Behavioral Risk Factor Surveillance System (BRFSS) study, a nationally representative probability-based sample of the US population. We included participants ≥18 years of age who were interviewed between 1999 and 2016. Using stratified weighting, we estimated the annual prevalence of participants with, and without a diagnosis of asthma, classified according to their body mass index (BMI), into: normal weight (18.5–25 kg/m^2^), overweight (25–30 kg/m^2^), or obese (>30 kg/m^2^). We calculated the annual odds of obesity among participants with vs. without asthma to assess if trends among individuals with asthma followed those of the general US population. Nominal regression analysis assessed the association between age, sex, race/ethnicity, and income with prevalence of obesity among participants with asthma.

**Results:**

Among the 543,574 BRSFF participants with asthma, the prevalence of overweight and obesity changed from 34.3% and 24.7% in 1999 to 28.8% and 41.1% in 2016, respectively. The odds ratio (OR) of obesity in patients with asthma compared to the general population without asthma, increased during the same period from 1.39 (95% confidence interval [CI]: 1.36–1.36) in 1999 to 1.75 (95% CI: 1.75–1.76) in 2016. Adjusted analysis showed that older (OR: 2.32, 95% CI: 2.32–2.33), Black (OR: 1.61, 95% CI: 1.61–1.61) and Hispanic (OR: 1.29, 95%. CI: 1.28–1.29) participants with asthma had higher rates of obesity.

**Conclusions:**

There has been a substantial increase in the prevalence of obesity among individuals with asthma in the last two decades, beyond what could be explained by general population trends. These results suggest that obesity is an increasing determinant of asthma morbidity and should be particularly targeted in minorities with asthma.

## Background

Asthma, a chronic condition characterized by airway inflammation and airflow obstruction, is one of the most prevalent diseases in the United State (US) and worldwide [1,2]. Obesity is a well-established risk factor for new onset asthma, asthma morbidity and poorer asthma-related quality of life [3,4,5,6]. Potential mechanisms underlying the relationship between obesity and poor asthma outcomes may include enhanced inflammation, increased level of oxidative stress, and decreased response to standard asthma medication (e.g., inhaled corticosteroids [ICS]) [4,23,24,26] increased prevalence of comorbidities such as gastro-esophageal reflux, sleep disorders, and depression associated with obesity [7,8,9], also worsen asthma control.

The prevalence of obesity in the US has increased steadily during the past 30 years, reaching an overall rate of 36.5% in the general population in 2014 [10,18,19,21,22]. However, less is known about the trajectory of obesity and overweight among individuals with asthma. This information is important to estimate the extent that obesity contributes to the burden of asthma and to develop policy interventions to curve the negative impact of obesity in this vulnerable group of patients.

In this study, we used data from the nationally representative U.S. Behavioral Risk Factor Surveillance System (BRFSS) to assess the trends of obesity and overweight in patients with asthma and to identify the subgroups of patients at higher risk of increased weight.

## Methods

The study was conducted with data from the Behavioral Risk Factor Surveillance System (BRFSS) study, a state-based random digit telephone survey that annually collects data from 50 US states, the District of Columbia and three US territories, regarding participants’ health behaviors and chronic conditions [11]. We included cross-sectional data from surveys conducted between 1999 and 2016 as presence of asthma was not consistently reported before this period. Additional details about the study can be found at the BRFSS webpage [11].

The study cohort consisted of adults ≥18 years of age who participated in one of the annual BRFSS cross sectional survey waves between 1999 and 2016. Participants with a diagnosis of asthma and symptomatic disease at the time of enrollment were classified as having active asthma. Participants with history of asthma but no current disease were included in the non-asthma comparison group. We calculated participants’ body mass index (BMI, in kilograms per meter square [kg/m^2^]) based on self-reported height and weight information. Using these data, participants were classified as underweight (<18.5 kg/m^2^), normal (18.5–25.0 kg/m^2^), overweight (25.0–30.0 kg/m^2^), or obese (>30.0 kg/m^2^).

We also collected BRFSS data on participants’ age (18–39 years, 40–64 years and ≥65 years), sex, race/ethnicity (withe non-Hispanic, Black non-Hispanic, Hispanic and others), income (<$25,000, $25,000–$49,999, $50,000–$74,999, and ≥$75,000 per year) and smoking status (current, former or never).

## Statistical Analysis

Using probability weighting based on sampling stratification, we estimated the annual prevalence (with 95% confidence intervals [CI]) of overweight and obesity among participants with and without asthma. Stratified analyses according to age, sex, and race/ethnicity were conducted to assess obesity trends in these subgroups. The odds ratio (OR) of overweight and obesity among asthmatic versus non-asthmatic participants was estimated for each year (1999 to 2016) to assess if obesity trends among participants with asthma followed those of the general US population.

We used nominal regression analysis to evaluate the adjusted association of age, sex, race/ethnicity, income and smoking status with prevalence of overweight and obesity in participants with asthma using data from the latest wave (2016) of the BRSFF survey.

Analyses were conducted with SAS (SAS, Cary, NC) and SPSS (IBM Corp., Armonk, NY) software using appropriate procedures to incorporate population sampling weights. The study was exempt by the Institutional Review Board of CEMIC Medical Center.

## Results

The study included 6,680,694 BRFSS participants, of these 543,574 (8.1%) reported active asthma. Overall, 212,161 (39.0%), 170,838 (31.4%) and 150,546 (27.7%) of participants with asthma were obese, overweight or had normal weight, respectively. Baseline characteristics of study participants according to weight categories are shown in Table 1.

**Table 1:** Baseline Sociodemographic Characteristics According to Weight Categories, Population Weighted Estimates.

Characteristic	Weight Category		

	Normal	Overweight	Obese

**Male, No. (%)**	31,827,956 (23.8%)	42,851,822 (32.1%)	58,896,749 (44.1%)
**Female, No. (%)**	62,165,911 (38.3%)	53,953,755 (33.2%)	46,162,572 (28.4%)
**Age years, No. (%)**			
**18–39**	47,558,508 (39.5%)	35,393,000 (29.4%)	37,497,406 (31.1%)
**40–64**	31,757,371 (23.9%)	42,988,056 (32.4%)	58,064,878 (43.7%)
**>65**	14,315,672 (28.6%)	18,045,688 (36.0%)	17,747,001 (35.4%)
**Race/Ethnicity, No. (%)**			
**White**	68,035,674 (32.7%)	66,755,181 (32.2%)	72,744,161 (35.1%)
**Black**	8,509,218 (22.4%)	10,894,045 (28.7%)	18,483,634 (48.8%)
**Hispanic**	9,394,316 (26.6%)	11,647,998 (33.0%)	14,219,855 (40.3%)
**Other**	7,173,563 (34.8%)	6,441,385 (31.2%)	7,015,238 (34.0%)
**Income, No. (%)**			
**<$25,000**	117,018,868 (61.9%)	28,559,870 (15.1%)	43,561,899 (23.0%)
**$25,000–$49,999**	20,270,850 (29.8%)	21,806,352 (32.0%)	25,978,122 (38.2%)
**$50,000–$74,999**	11,421,737 (30.2%)	12,864,432 (34.0%)	13,522,880 (35.8%)
≥**$75,000**	21,411,414 (35.1%)	21,894,226 (35.9%)	17,737,083 (29.1%)
**Smoking History, No. (%)**			
**Current**	23,998,681 (34.1%)	21,415,932 (30.5%)	24,837,196 (35.4%)
**Former**	19,776,731 (5.4%)	26,296,771 (7.2%)	320,008,200 (87.4%)
**Never**	49,284,550 (32.2%)	48,165,513 (31.5%)	55,591,036 (36.3%)

**Figure 1:**
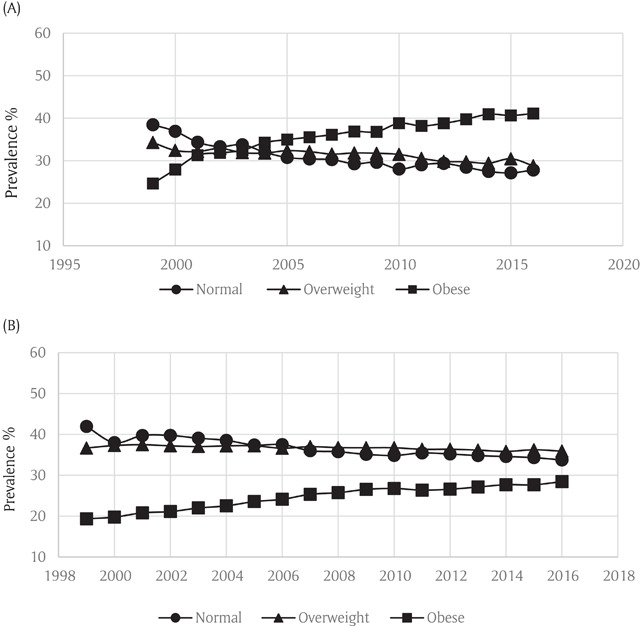
**(A)** Weight Trends in Participants with Asthma in the Behavioral Risk Factor Surveillance System. **(B)** Weight Trends in Individuals without Asthma in the Behavioral Risk Factor Surveillance System.

**Figure 2:**
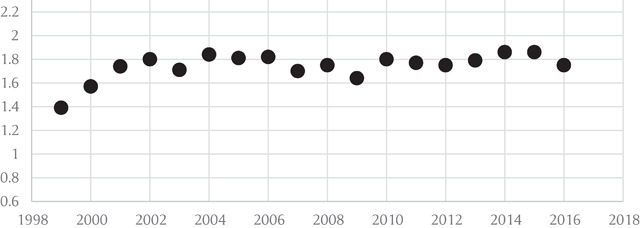
Trends of Odds Ratio for Obesity in Asthmatics vs Non-Asthmatics in the Behavioral Risk Surveillance System.

The prevalence of overweight and obesity among participants with asthma increased from 34.3% and 24.7% in 1999 to 28.8% and 41.1% in 2016, respectively (Figure 1A). Trends of obesity and overweight among non-asthma BRFSS participants from 1999 to 2016 are shown in Figure 1B. Overall, 36.7% and 19.4% of non-asthmatic participants were overweight and obese in 1999 compared to 35.9% and 28.4% in 2016. The odds ratio of obesity in participants with versus without asthma increased from 1.36 (95% CI: 1.38–1.39) in 1999 to 1.75 (95% CI: 1.75–1.76) in 2016 (Figure 2), showing that obesity rates increased faster among asthma participants than that in the general population.

**Figure 3:**
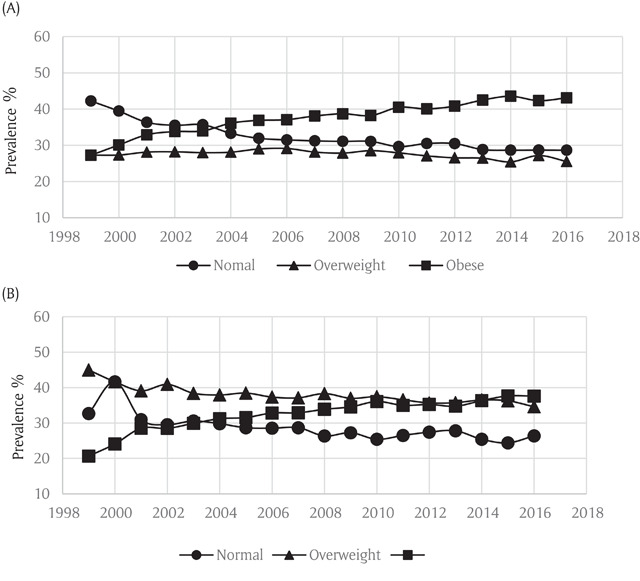
**(A)** Weight Trends in Asthmatic Females in the Behavioral Risk Factor Surveillance System. **(B)** Weight Trends in Asthmatics Males in the Behavioral Risk Factor Surveillance System.

**Figure 4:**
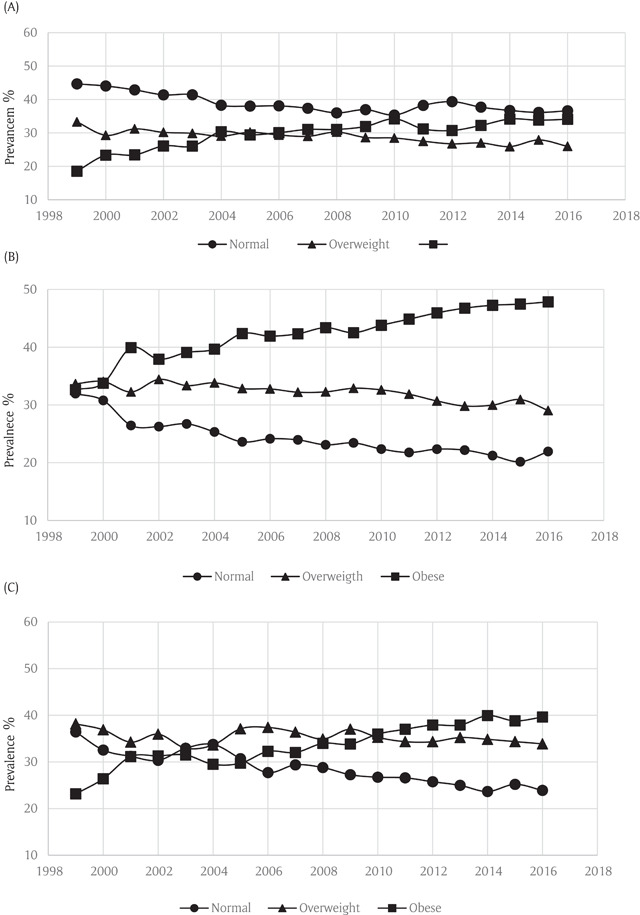
**(A)** Weight Trends in 18–39 years old Asthmatics in the Behavioral Risk Factor Surveillance System. **(B)** Weight Trends in 40–64 years old Asthmatics in the Behavioral Risk Factor Surveillance System. **(C)** Weight Trends in ≥65 Asthmatics in the Behavioral Risk Factor Surveillance System.

**Figure 5:**
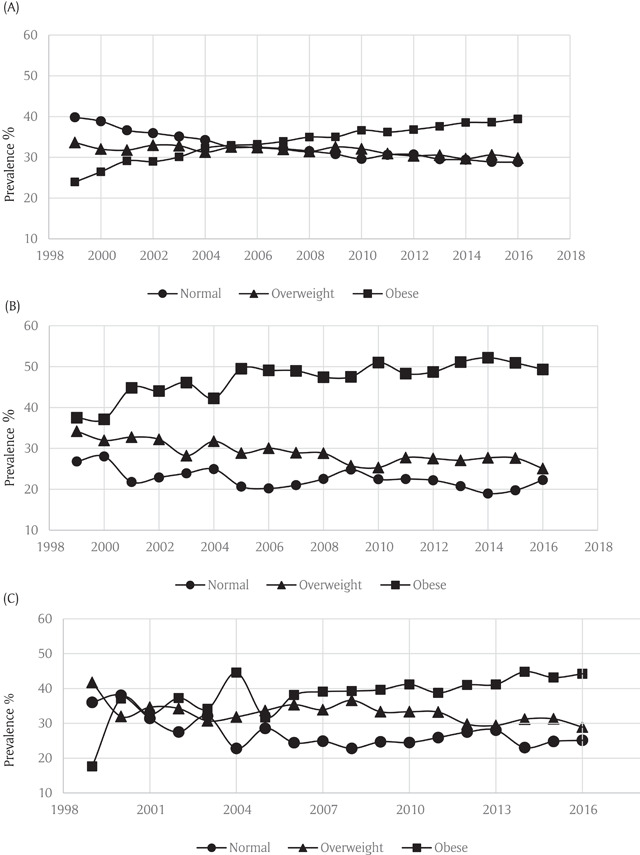
**(A)** Weight Trends in White Asthmatics in the Behavioral Risk Factor Surveillance System. **(B)** Weight Trends in Black Asthmatics in the Behavioral Risk Factor Surveillance System. **(C)** Weight Trends in Hispanic Asthmatics in the Behavioral Risk Factor Surveillance System.

Analyses stratified by sex showed similar trends in both men and women. Rates of obesity increased from 27.3% to 43.1% among females and 20.7% to 37.6% in males during the study period (Figure 3). All age groups showed an increase in obesity rates during the study period (Figure 4). Stratification by race/ethnicity also showed a pattern of increased obesity rates among all groups. Rates of obesity rose from 23.9% to 39.4% in non-Hispanic whites, from 37.5% to 49.3% in blacks and from 17.6% to 34.8% in Hispanics (Figure 5).

In the nominal regression analysis, we found that asthma participants ≥65 years of age had higher odds of being overweight (OR: 1.90, 95% CI: 1.89–1.91) compared to those 18–39 years of age. Black and Hispanic participants also had increased odds of being overweight (OR: 1.20, 95% CI: 1.20–1.21 and OR: 1.30, 95% CI: 1.29–1.30, respectively) as did former smokers (OR: 1.27, 95% CI: 1.26–1.27). Independent predictors of overweight included age 40–64 years (OR: 2.32, 95% CI: 2.32–2.33) and ≥65 years (OR: 1.58, 95% CI: 1.57–1.58) compared with age 18–39 years, blacks (OR: 1.61, 95% CI: 1.61–1.61), Hispanics (OR: 1.29, 95%. CI: 1.28–1.29) compared to whites and former smokers (OR: 1.33, 95% CI: 1.32–1.33) compared to never smokers (Table 2).

**Table 2:** Association between Sociodemographic Factors and Prevalence of Obesity and Overweight among Asthma Participants in the Behavioral Risk Factor Surveillance System (2016 Wave).

Variable	OR of Overweight vs. Normal Weight (95% CI)	OR of Obese vs. Normal Weight (95% CI)

**Female, No. (%)**	0.65 (0.65–0.65)	0.98 (0.98–0.98)
**Age, in years, No. (%)**		
**18–39**	Reference	Reference
**40–64**	1.80 (1.80–1.81)	2.32 (2.32–2.33)
**>65**	1.90 (1.89–1.91)	1.58 (1.57–1.58)
**Race/Ethnicity, No. (%)**		
**White**	Reference	Reference
**Black**	1.20 (1.20–1.20)	1.61 (1.61–1.61)
**Hispanic**	1.30 (1.29–1.30)	1.29 (1.28–1.29)
**Other**	0.72 (0.72–0.72)	0.72 (0.72–0.72)
**Income, No. (%)**		
**<$25,000**	Reference	Reference
**$25,000–$49,999**	0.99 (0.99–1.00)	0.87 (0.87–0.87)
**$50,000–$74,999**	1.14 (1.14–1.14)	0.84 (0.83–0.83)
≥**$75,000**	0.92 (0.91–0.92)	0.52 (0.51–0.52)
**Smoking History, No. (%)**		
**Never**	Reference	Reference
**Current**	0.93 (0.93–0.93)	0.81 (0.81–0.81)
**Former**	1.27 (1.26–1.27)	1.33 (1.32–1.33)

OR: odds ratio, CI: confidence interval.

## Discussion

Using a nationally representative US population-based data set we found a substantial increase in the rates of obesity among individuals with asthma during the last two decades. These trends were consistent among different sociodemographic groups and were greater than those observed in the general US population. Our findings suggest that obesity is a growing problem in individuals with asthma and may have an increasing role as a cause of lack of control and poorer quality of life. These results could guide management decisions and the design of interventions to address this major public health problem.

The relationship between obesity and asthma is well recognized and confirmed in multiple epidemiologic studies and meta-analyses [3,5,12,13]. Increased weight is not only associated with greater incidence of asthma but is also related to an attenuated response to ICS therapy, worse disease control, poorer quality of life and increased risk of asthma-related resource utilization [4,8,14]. The mechanisms underlying the relationship between obesity and asthma severity are not fully understood but may include difference in inflammatory endotypes, changes in oxidative stress, increased prevalence of comorbid conditions such as gastroesophageal reflux and obstructive sleep asthma, and potentially differences in self-management behaviors [3,23,25,26]. Given the major impact of obesity on the outcomes of patients with asthma, there is a growing interest in testing the effectiveness of weight loss interventions as well as integrated behavioral programs to concomitantly address these conditions [15].

Obesity has become a major health problem worldwide. In the US, the prevalence of obesity in the general adult population has reached a peak rate of 36.5%. There are several studies that show how obesity increases the prevalence of asthma [3,5,12,13,16,20]. Asthma has a prevalence rate in the general US population of 8.8%. However, rates of asthma are 11.1% among obese adults compared to 7.1% in lean individuals and 7.8% among overweight adults [16]. According to the Centers for Disease Control and Prevention (CDC), the rate of obesity in adults with current asthma in 2010 was almost 39% and significantly higher than the rate among adults without the disease (27%). Moreover, the CDC report showed that rates of overweight and obesity substantially differ from state to state with the highest rates being clustered in the Midwest and South of the US. The literature is inconclusive regarding the sociodemographic factors that combine with obesity to drive asthma prevalence higher. While some studies have found a greater effect in women, others have shown a similar increase in obesity-related asthma in individuals of both genders [12,27]. In contrast, studies have consistently demonstrated increased rates of obesity among black and Hispanic individuals with asthma [12,16,27].

The current study has strengths and weaknesses that are worth mentioning. The BRFSS is a population-based survey that samples individuals from the entire US; thus, the generalizability of our results should be strong. The large sample size of the BRFSS, combined with consistent information about BMI and asthma over an 18-year period allowed us to obtain precise estimates of the trends of obesity among US adults with asthma. However, the BRFSS only includes self-reported data about asthma and BMI, which may be subject to reporting bias. Additionally, a recent Canadian study showed that a large percentage of individuals with self-reported physician diagnosis of asthma did not have the disease upon more detailed testing [17]. Lack of consistent information about lung function testing or asthma control in some of the BRFSS waves limited our ability to assess whether population changes in obesity were associated with poorer disease control. Additionally, our study did not evaluated trends of obesity in children with asthma.

In summary, our study showed a consistent increase in the prevalence of obesity among US adults with asthma. While changes were observed across multiple populations, certain groups, such as racial and ethnic minorities, appear to be at higher risk of obesity. This observation may explain, in part, the worse asthma outcomes experienced by individuals from these populations. Our findings are important for planning policy interventions to curve the obesity epidemic in patients with asthma.
